# Nemaline myopathy in newly diagnosed systemic lupus erythematosus and Sjögren’s overlap syndrome complicated by macrophage activation syndrome

**DOI:** 10.1186/s41927-022-00246-2

**Published:** 2022-03-15

**Authors:** Christina Vogel, Poonam Manwani, Marcia E. Cornford, Emil R. Heinze

**Affiliations:** 1 UCLA-Olive View Internal Medicine Program, Department of Medicine, Olive View-UCLA Medical Center, 14445 Olive View Drive, 2B182, Sylmar, CA 91342 USA; 2 UCLA-Olive View Rheumatology Program, Division of Rheumatology, Olive View-UCLA Medical Center, 14445 Olive View Drive, 2B182, Sylmar, CA 91342 USA; 3 Department of Pathology, Harbor-UCLA Medical Center, 1000 W Carson St, Torrance, CA 90502 USA

**Keywords:** Nemaline myopathy, Systemic lupus erythematosus, Sjögren’s syndrome, Macrophage activation syndrome, Sporadic late-onset nemaline myopathy

## Abstract

**Background:**

Nemaline myopathies are congenital or acquired muscle disorders that typically present in childhood but can occasionally occur in adults with underlying malignant, infectious or autoimmune disorders. There is a great genetic heterogeneity as well as clinical variability among the disease.

**Case presentation:**

Here, we present a case of nemaline myopathy in a young woman who was newly diagnosed with systemic lupus erythematosus (SLE) and Sjögren’s overlap syndrome complicated by macrophage activation syndrome (MAS). She had no personal or family history of myopathy and was reporting progressive thigh weakness. A muscle biopsy revealed type 1 myofiber predominance with granular material in atrophic myocytes consistent with nemaline myopathy. Her symptoms markedly improved with immunotherapy for her SLE and MAS supporting the diagnosis of sporadic late-onset nemaline myopathy (SLONM) associated with her autoimmune disease.

**Conclusions:**

SLONM is a type of nemaline myopathy that presents in adults and can occasionally be associated with autoimmune disease. In these cases, treatment of the underlying disorder with immunosuppression appears to improve symptoms of myopathy.

**Supplementary Information:**

The online version contains supplementary material available at 10.1186/s41927-022-00246-2.

## Background

Nemaline myopathy is a heterogeneous group of muscle disorders characterized by rod-shaped bodies on muscle biopsy. It most commonly occurs as a congenital myopathy of variable expression with clinical presentations ranging from mild, non-progressive, to more severe phenotypes, which can be fatal within the first year of life. Less commonly, it presents as an adult-onset form called sporadic late-onset nemaline myopathy (SLONM). This form can be associated with monoclonal gammopathy of indetermined significance (MGUS), multiple myeloma and human immunodeficiency virus (HIV) and usually presents in adults after age 40 [1–4]. It typically affects the proximal musculature and can lead to respiratory insufficiency [1–4].

Here, we present a case of newly diagnosed systemic lupus erythematosus (SLE) and Sjögren’s overlap syndrome complicated by macrophage activation syndrome (MAS) in a previously healthy young woman who was also found to have nemaline myopathy. To our knowledge, there are only two case reports describing adult-onset nemaline myopathy in patients with SLE [5, 6] and one case report of nemaline myopathy in a patient with primary Sjögren’s syndrome [7].

## Case presentation

**Fig. 1:**
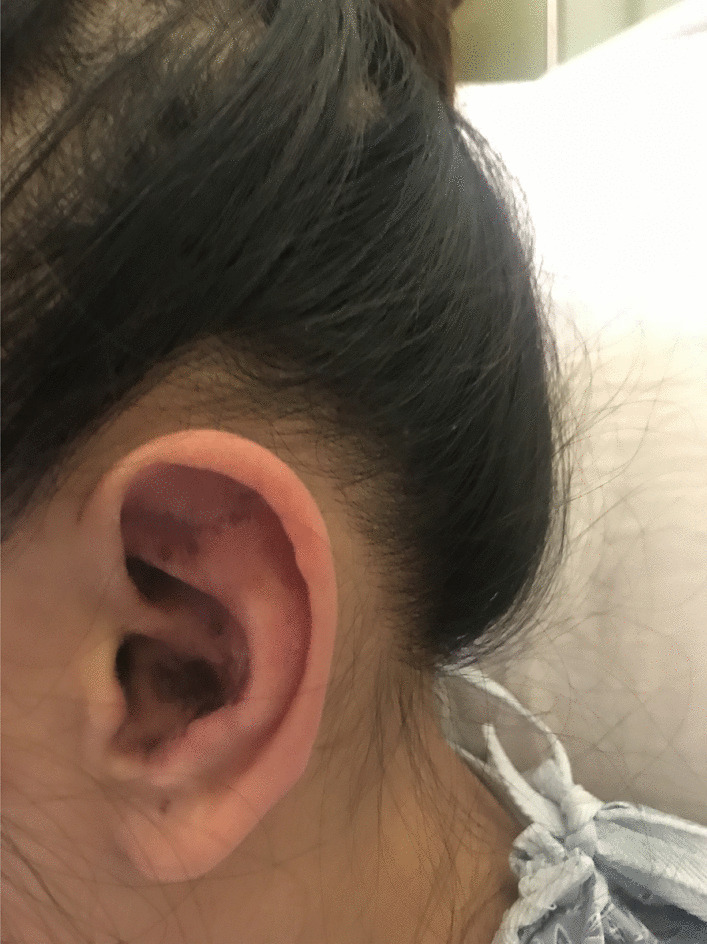
Left conchal bowl and antihelix with well demarcated violaceous to hyperpigmented patches. Scalp with round patches of non-scarring alopecia

**Fig. 2:**
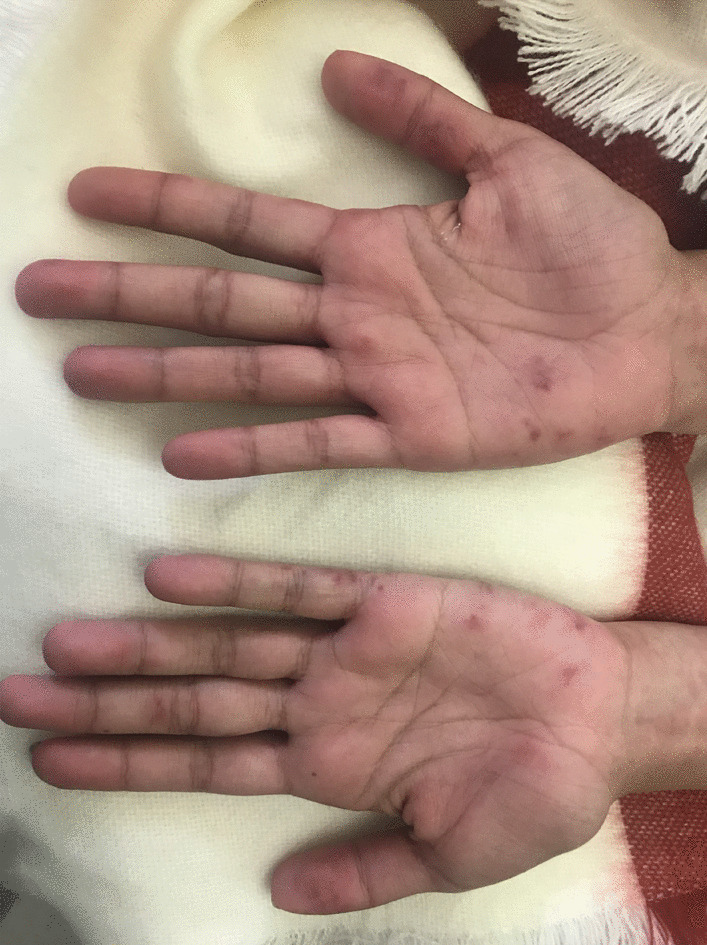
Erythematous to violaceous papules on palmar surface of the first digit and hypothenar and a few additional scattered erythematous lesions

A 22-year-old female with no past medical history presented to our hospital with a rash for several months that started on her face, then spread to her fingertips, the tips of her toes and her scalp (Figs. 1, 2). She also had some upper lip swelling without associated dyspnea, dysphonia or dysphagia. The patient reported feeling more fatigued for the last several months, subjective fevers and easy fatigability of her proximal lower extremities. She had been prescribed minocycline one month prior to her presentation for presumed acne by an outside provider with minimal improvement in her rash. Her physical examination was notable for an ill-defined, erythematous to violaceous, papular rash involving the nasal bridge, cheeks, ears, scalp, her bilateral upper and lower extremities and multiple dime-sized hairless lesions on the scalp (Figs. 1, 2). She was noted to have an edematous upper lip as well as oral and nasal ulcers and an enlarged cervical lymph node. Strength and sensation were noted to be intact in all four extremities with an objective muscle strength of 5/5 in all muscle groups and without any joint tenderness or swelling. Her laboratory workup showed pancytopenia with a hemoglobin of 11.9 g/dL (ref 12.0–14.6), a white cell count of 2.2 K/cumm (ref 4.5–10.0) and a platelet count of 120 K/cumm (ref 160–360). Transaminases were markedly elevated and continued to rise after admission (AST up to 3205 U/L (ref 15–41) and ALT up to 1058 U/L (ref 14–54). CK was mildly elevated at 239 U/L (ref 26–174). Lipase was found to be increased > 4400 U/L (ref 22–51). Additional laboratory workup revealed a positive ANA with a titer of 1:2560 and a speckled ANA pattern, positive anti-Smith antibodies, positive SSA, positive SM/RNP with negative anti-dsDNA antibodies. C3 and C4 were found to be decreased at 21 mg/dL (ref 90–180) and 5.5 mg/dL (ref 10–40) respectively. HIV testing was negative. The patient was found to meet the 2019 ACR/EULAR classification criteria for SLE [8] (positive ANA, fever, acute cutaneous lupus, oral ulcers, nonscarring alopecia, leukopenia, thrombocytopenia, anti-Sm antibodies, and low complement levels) and the 2016 ACR/EULAR classification criteria for Sjögren’s [9] (positive anti-SSA and positive Schirmer’s test), so a diagnosis of SLE overlap with Sjögren’s syndrome was made and the patient was started on hydroxychloroquine 200 mg daily.

During her hospital course, she spiked a fever and was started on empiric antibiotics. A thorough infectious workup remained negative. She underwent a CT of the neck which demonstrated numerous enlarged lymph nodes of variable size bilaterally with enlarged bilateral adenoids, raising concern for lymphoma. A CT of the chest, abdomen and pelvis showed bilateral axillary lymphadenopathy, hepatic steatosis and mild hepatomegaly. A fine needle aspiration lymph node biopsy of the enlarged cervical lymph node was performed and showed a low cellular specimen comprised of a polymorphous lymphoid population with scattered atypical cells. She was found to have a ferritin > 11,000 ng/ml (ref 5–204) and an IL-2R of 4995 pg/ml (ref 532–1891). A bone marrow biopsy resulted with hemophagocytosis consistent with hemophagocytic lymphohistiocytosis (HLH). The patient was started on dexamethasone 10 mg/m2 daily for macrophage activation syndrome (MAS). Cyclosporine 75 mg twice daily and mycophenolate mofetil 500 mg twice daily were added as steroid-sparing agents.

**Fig. 3:**
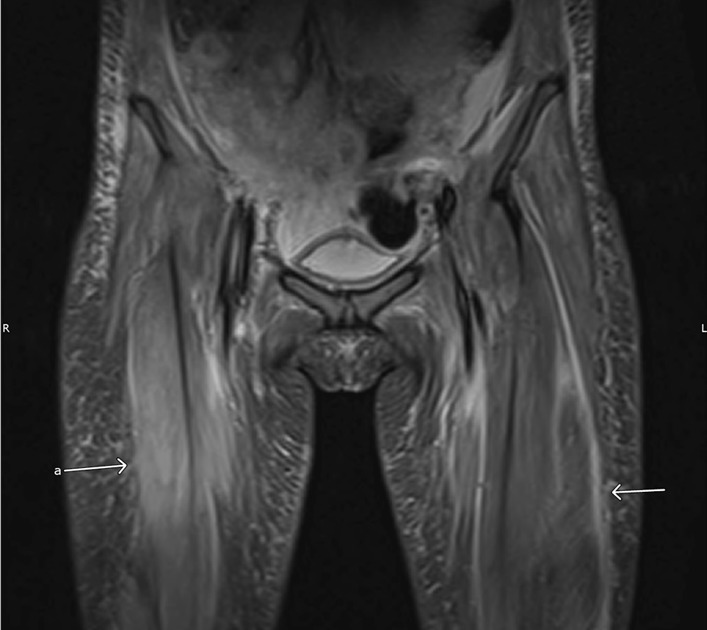
MRI Pelvis T1 fluid sensitive STIR sequence with arrows pointing to abnormal muscle signal of the muscles of quadriceps femoris indicating myoedematous changes

**Fig. 4:**
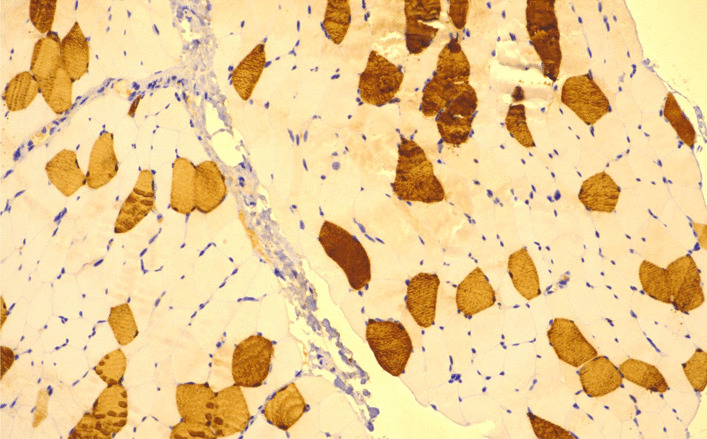
Light microscopy of quadriceps femoris muscle biopsy showing fiber type 1 predominance (× 20, ATPase stain. Images obtained with Nikon Eclipse Ci with Plan Apo lenses using Cellsens imaging software. Definition 1600 × 1200 pixels)

**Fig. 5:**
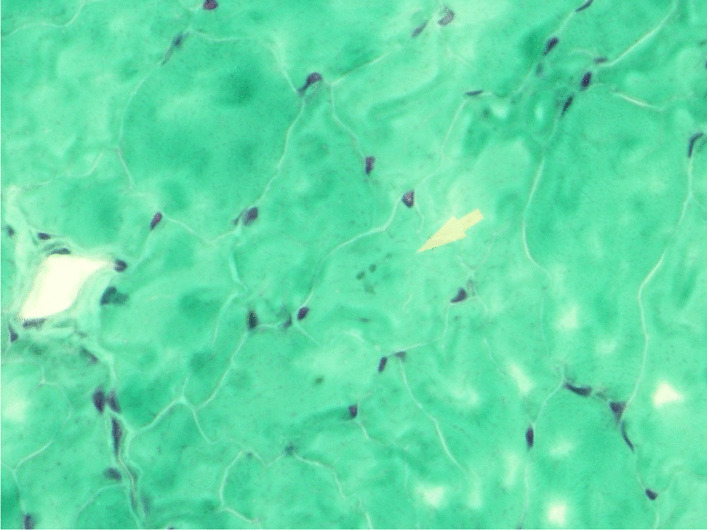
Light microscopy of quadriceps femoris muscle biopsy with arrow pointing to nemaline bodies (× 60, Gomori trichrome stain. Images obtained with Nikon Eclipse Ci with Plan Apo lenses using Cellsens imaging software. Definition 1600 × 1200 pixels)

**Fig. 6:**
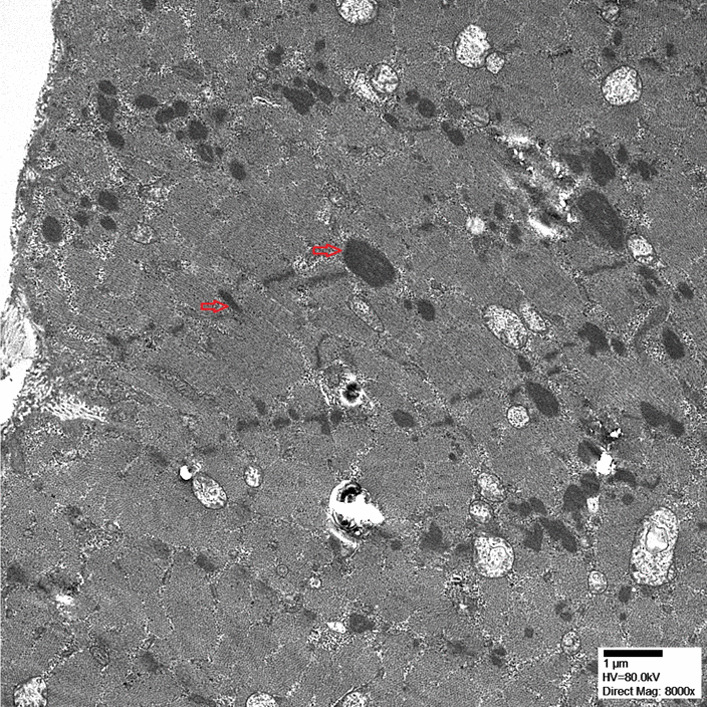
Electron microscopy of quadriceps femoris muscle biopsy showing occasional early dense bodies in continuity with the Z-disc and occasional dense rods reminiscent of nemaline bodies (× 8000)

An MRI of the pelvis revealed abnormal muscle signal on fluid-sensitive (STIR, short tau inversion recovery) sequences indicating edematous changes of the muscles of the pelvis and proximal thighs (Fig. 3). A quadriceps femoris muscle biopsy was performed and showed nemaline rod myopathy with fiber type disproportion and type 1 myofiber predominance (Figs. 4, 5). Light microscopy was followed by electron microscopy to confirm the findings of nemaline rods (Fig. 6). A comprehensive genetic neuromuscular disorder panel was negative for any known pathogenic mutations.

Throughout the hospital course the patient experienced marked improvement in her strength, fatigue, rash, cytopenias, transaminases, lipase, ferritin, CK and aldolase. We were finally able to discharge her home on hydroxychloroquine, mycophenolate mofetil, cyclosporine and a dexamethasone taper. On follow-up several months after discharge, her symptoms continued to improve with her strength back to her baseline.

## Discussion and conclusion

Nemaline myopathies are among the most frequent congenital myopathies. They are hallmarked by rod- or thread-shaped cytoplasmic bodies and named after *nema*, the Greek word for thread [10]. Nemaline bodies are derived from degenerated Z-bands and stain positive on immunohistochemistry for Z-band proteins, such as alpha-actinin and myotilin [1]. They are visualized as fine cytoplasmic aggregates on light microscopy and can be confirmed by high electron density similar to Z-bands on electron microscopy [1]. Given their association with plasma cell dyscrasias, HIV and autoimmune disorders, immune dysregulation likely plays a key role in their etiology [6, 7]. Another possible explanation for their appearance includes an unrecognized viral infection as a possible trigger [6, 7].

There is a great genetic heterogeneity as well as clinical variability among the disease. The majority of congenital nemaline myopathies present in infancy or childhood, although some have been reported to be relatively asymptomatic until early adulthood. Sporadic late-onset nemaline myopathies (SLONM) on the other hand typically present in adults after the age of 40 and are commonly associated with MGUS and multiple myeloma and can less frequently be associated with HIV [1, 2], although an association with autoimmune disorders has also been described [5–7]. They are sometimes referred to as “myopathies with rods” to distinguish them from congenital nemaline myopathies [11].

Congenital nemaline myopathies are characterized by hypotonia and a predominantly proximal muscle weakness involving the face, neck flexors and proximal extremities [12]. Respiratory symptoms are frequently noted and range from asymptomatic restriction seen on pulmonary function testing to sudden respiratory failure in infancy [12]. Treatment is generally supportive.

Sporadic late-onset nemaline myopathy (SLONM) typically presents with weakness of the proximal upper and lower extremities, dyspnea, dysarthria and dysphagia [4]. SLONM associated with MGUS tends to have a more severe course with rapid progression to respiratory failure compared with SLONM associated with HIV [4]. Muscle pathology typically shows fiber type disproportion and type 1 fiber predominance, as seen in our patient [11]. Treatments that have been suggested for SLONM with MGUS are IVIG as well as anti-plasma cell dyscrasia therapy including autologous stem-cell transplantation following high-dose melphalan [1, 3]. HIV-associated nemaline myopathy on the other hand has been reported to respond to immunosuppressive therapy [4]. It is unclear whether SLONM and HIV-NM represent two separate disease entities.

To our knowledge, there have only been two other case reports published describing adult-onset nemaline myopathy in patients with SLE. One of the patients had symptoms of muscle weakness since infancy which was found to be secondary to congenital nemaline myopathy [5]. The other case was thought to be secondary to SLONM and improved with rituximab and cyclophosphamide [6]. Another case report delineates a case of SLONM in a patient with primary Sjögren’s syndrome with symptoms responding to steroids and IVIG [7].

The case of SLE associated with SLONM was described in a 41-year-old woman who was diagnosed with SLE six years prior. She was suffering from significant muscle weakness since her diagnosis of SLE, which progressed until she was unable to walk without bilateral support. CK level and serum electrophoresis were normal; HIV testing was negative. A deltoid muscle biopsy revealed myofibers with nemaline rods, which were confirmed on electron microscopy. Her weakness eventually responded to rituximab and cyclophosphamide which were started for her concurrent lupus nephritis [6].

The case of Sjögren’s syndrome and SLONM was reported in a 58-year-old woman who presented with progressive muscle weakness that progressed over the course of two years causing severe difficulty walking. She had a normal serum CK and negative HIV testing. A modified Gomori trichrome stain of a quadriceps muscle biopsy showed rods in more than half of the muscle fibers, many of them irregular and punctuate, which were confirmed on EM. Fiber type proportions were noted to be normal. The patient’s symptoms improved with steroids and IVIG [7].

Our patient presented with subjective weakness confined to her bilateral proximal lower limbs. Her personal and family histories were negative for any kind of myopathies and with absence of known consanguinity. An MRI of the pelvis showed abnormal muscle signal on fluid-sensitive sequences indicating edema of the muscles of the pelvis and proximal thighs that appeared most severe in the gluteus muscles. A quadriceps biopsy revealed type 1 myofiber predominance with granular material in atrophic myocytes consistent with nemaline myopathy. Her symptoms markedly improved after initiation of immunotherapy for SLE and MAS with hydroxychloroquine, dexamethasone, cyclosporine and mycophenolate mofetil. All of these features make a diagnosis of SLONM associated with SLE and Sjögren’s syndrome highly likely.

Limitations of our case report include the lack of workup for MGUS/multiple myeloma including serum/urine electrophoresis and immunofixation.

In conclusion, SLONM is a type of nemaline myopathy that presents in adults and can occasionally be associated with autoimmune disease. In these cases, treatment of the underlying disorder with immunosuppression can improve symptoms of myopathy.

## Supplementary Information

**Additional file 1.** Timeline of case patient. This timeline depicts the presentation of the case patient with physical examination, imaging and laboratory findings.

## Data Availability

The case clinical data used to support the findings of this study are included within the article.

## Abbreviations

SLE: Systemic lupus erythematosus
MAS: Macrophage activation syndrome
SLONM: Sporadic late-onset nemaline myopathies
MGUS: Monoclonal gammopathy of undetermined significance
HIV: Human immunodeficiency virus
ANA: Antinuclear antibody
CT: Computed tomography
MRI: Magnetic resonance imaging
STIR: Short tau inversion recovery
HLH: Hemophagocytic lymphohistiocytosis
CK: Creatine kinase
IVIG: Intravenous immune globulin
