# Supplementary material for: Nemaline myopathy in newly diagnosed systemic lupus erythematosus and Sjögren’s overlap syndrome complicated by macrophage activation syndrome
Source: BMC Rheumatol. 2022 Mar 15;6:21. doi: 10.1186/s41927-022-00246-2 (PMC8922735; doi:10.1186/s41927-022-00246-2)
Supplement: Supplementary file 1 — Additional file 1. Timeline of case patient. This timeline depicts the presentation of the case patient with physical examination, imaging and laboratory findings. [file 41927_2022_246_MOESM1_ESM.docx]

**Case Report - Timeline**

| Date |  |
| --- | --- |
| One month prior to admission | Patient presented to outside provider for facial rash, was prescribed minocycline for presumed acne. |
| Two days prior to admission | Patient presented to our urgent care clinic for rash now extending to scalp, palms and feet. Reporting fatigue. Extensive lab workup sent including liver function tests, infectious and autoimmune labs. |
| Day of admission | Presented for follow-up. Reporting subjective fevers and easy fatiguablity of the thighs. Noted to have pancytopenia (hemoglobin of 11.9 g/dL (ref 12.0-14.6), a white cell count of 2.2 K/cumm (ref 4.5-10.0) and a platelet count of 120 K/cumm (ref 160-360)) and transaminitis (AST 112 (ref 15-41), ALT 63 (ref 14-54)). CK elevated to 239 U/L (ref 26-174). Anti-smith antibodies positive, SM/RNP antibodies positive, anti-dsDNA antibodies negative. Admission for further workup. |
| Hospital Day 2 | Noted to have fever overnight. Started on empiric antibiotics. Infectious workup sent. |
| Hospital Day 3 | C3 and C4 resulted low at 21 mg/dL (ref 90-180) and 5.5 mg/dL (ref 10-40). Rheumatology consulted, started hydroxychloroquine for SLE.  GI consulted for elevated liver enzymes. CT Neck showing cervical lymphadenopathy, CT Chest/Abdomen/Pelvis showing axillary lymphadenopathy, hepatomegaly. |
| Hospital Day 4 | Hematology/Oncology consulted. US-guided cervical lymph node biopsy. Bone marrow biopsy. ANA resulted high positive (titer 1:2560), ferritin >11,000 (ref 5-204). Started dexamethasone 10mg/m2 daily given concern for MAS. |
| Hospital Day 5 | AST peaked at 3,205 U/L (ref 15-41) and ALT peaked at 1,058 U/L (ref 14-54). |
| Hospital Day 6 | Bone marrow biopsy resulted with hemophagocytosis. Skin biopsy done. Rash improving. |
| Hospital Day 11 | Cyclosporine 75mg BID added as steroid-sparing agent. MRI Pelvis done, showing nonspecific myositis in the pelvis and proximal thighs. |
| Hospital Day 13 | Mycophenolate mofetil 500mg BID added. |
| Hospital Day 15, Day of Discharge | Marked improvement in liver enzymes (AST 49 (ref 15-41), ALT 97 (ref 14-54)) and inflammatory markers (ferritin 390 (ref 5-204)). Patient feeling much improved. Discharged home with dexamethasone taper. |
| Two days after discharge | Left thigh muscle biopsy done. |
| Eleven days after discharge | Muscle biopsy resulted with nemaline rod myopathy with congenital fiber type disproportion (type 1 myofiber predominant). |
| Six weeks after discharge | Rheumatology follow up. Patient reporting marked improvement in strength, now back to baseline. |
